# All-optical modulator with photonic topological insulator made of metallic quantum wells

**DOI:** 10.1515/nanoph-2024-0197

**Published:** 2024-06-26

**Authors:** Haiteng Wang, Junru Niu, Qiaolu Chen, Sihan Zhao, Hua Shao, Yihao Yang, Hongsheng Chen, Shilong Li, Haoliang Qian

**Affiliations:** Interdisciplinary Center for Quantum Information, State Key Laboratory of Modern Optical Instrumentation, College of Information Science and Electronic Engineering, 12377 Zhejiang University, Hangzhou 310027, China; ZJU-Hangzhou Global Science and Technology Innovation Center, Key Lab of Advanced Micro/Nano Electronic Devices & Smart Systems of Zhejiang, 12377 Zhejiang University, Hangzhou 310027, China; International Joint Innovation Center, ZJU-UIUC Institute, 12377 Zhejiang University, Haining 314400, China; Interdisciplinary Center for Quantum Information, State Key Laboratory of Silicon and Advanced Semiconductor Materials, and Zhejiang Province Key Laboratory of Quantum Technology and Device, School of Physics, 12377 Zhejiang University, Hangzhou 310058, China

**Keywords:** photonic topological insulator, all-optical modulator, metallic quantum well, Kerr nonlinearity

## Abstract

All-optical modulators hold significant prospects for future information processing technologies for they are able to process optical signals without the electro-optical convertor which limits the achievable modulation bandwidth. However, owing to the hardly-controlled optical backscattering in the commonly-used device geometries and the weak optical nonlinearities of the conventional material systems, constructing an all-optical modulator with a large bandwidth and a deep modulation depth in an integration manner is still challenging. Here, we propose an approach to achieving an on-chip ultrafast all-optical modulator with ultra-high modulation efficiency and a small footprint by using photonic topological insulators (PTIs) made of metallic quantum wells (MQWs). Since PTIs have attracted significant attention because of their unidirectional propagating edge states, which mitigate optical backscattering caused by structural imperfections or defects. Meanwhile, MQWs have shown a large Kerr nonlinearity, facilitating the development of minimally sized nonlinear optical devices including all-optical modulators. The proposed photonic topological modulator shows a remarkable modulation depth of 15 dB with a substantial modulation bandwidth above THz in a tiny footprint of only 4 × 10 µm^2^, which manifests itself as one of the most compact optical modulators compared with the reported ones possessing a bandwidth above 100 GHz. Such a high-performance optical modulator could enable new functionalities in future optical communication and information processing systems.

## 1 Introduction

All-optical modulations have emerged as one of the most essential ingredients for integrated photonics due to their ultrafast operation speed [1], [2], [3], [4], [5], [6], [7], [8], [9], [10], [11], [12], [13]. Material systems with a relatively large Kerr effect in various spatial configurations, such as the Mach–Zehnder interferometer (MZI) [14], [15], [16] and the ring-assisted MZI [17], [18], have commonly been utilized for the all-optical modulators. Nevertheless, these all-optical modulators suffer from (i) a large device footprint due to the weak optical nonlinearities of the conventional material systems – which limits their application for dense photonic integrated circuits, and (ii) a strong optical backscattering owing to the unavoidable structure defects of the commonly-used device geometries – which results in a small modulation depth. Therefore, new photonic platforms with a robust optical transport and a small feature size are in urgent need for the next-generation high-performance all-optical modulators.

Photonic topological insulators (PTIs) have recently emerged as an intriguing photonic platform that supports a robust optical transport. They are characterized by the presence of unidirectional propagating edge modes which exhibit remarkable robustness against imperfections or defects, thus effectively preventing optical backscattering [19]. Such a robustness feature has enabled an unprecedented evolution in various optical signal processing processes such as signal non-reciprocal transport [20], [21], [22], routing [23] and isolation [24]. As a result, numerous innovative photonic devices have been conceptualized and demonstrated ranging from reflection-free sharply waveguides [25], [26], spin-polarized switches [27], [28], to non-reciprocal circulators and travelling wave amplifiers [29], [30]. However, limited also by the weak optical nonlinearities of commonly-used materials, PTIs based all-optical modulators have not been fully explored.

Many recent efforts have made use of metallic quantum wells (MQWs) to achieve giant ultrafast optical nonlinearities due to the quantum size effect [31], [32], [33], [34]. In this work, we propose and demonstrate an on-chip ultrafast all-optical modulator by using PTIs made of these MQWs. The MQW-based all-optical PTI modulator shows a remarkable modulation depth of 15 dB with a substantial modulation bandwidth above THz [35], and its size is only 4 × 10 µm^2^ – a great advantage for large-scale integration (LSI) photonic integrated circuits. Moreover, the device size can be further reduced to 1.3 × 10 μm^2^ at the 3-dB modulation depth with a transmission of −1.68 dB. Such an all-optical modulator holds significant promise for advancing the field of nonlinear topological photonics and expanding its applicability across diverse domains, including optical communications, microwave photonics, and quantum information processing.

## 2 Results

**Figure 1:**
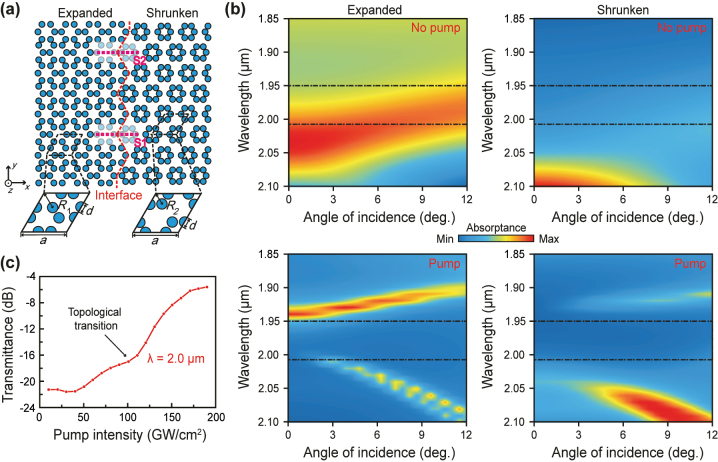
MQW-based PTI used for the proposed all-optical modulator. (a) Honeycomb lattice of cylindrical pillars made of TiN/Al_2_O_3_ MQWs with two different unit geometries: the expanded one with *a*/*R*_1_ = 2.75 and the shrunken one with *a*/*R*_2_ = 3.65, where the lattice period *a* = 1.58 µm, and the distances between adjacent cylindrical pillars of two different unit geometries *R*_1_ = 0.57 µm and *R*_2_ = 0.43 µm. In both cases, the height of the pillar *h* = 1 µm, and its diameter *d* = 0.37 µm. (b) Spectra for expanded and shrunken PTIs without (the upper panels) and with (the lower panels) laser pumping. The horizontal axis is the incidence angle of the incident signal beam onto the PTI waveguide, while the angle of the pump beam remains 45° with respect to the PTI waveguide. Color encodes the magnitude of absorptance (*A*) of these PTIs for *p*-polarized incident light. The dashed squares mark the band gap of PTI. Simulation details are presented in Supplementary Material Section 2. (c) Transmittance *T* = *P*_*y*_S1_/*P*_*y*_S2_ at the wavelength of 2.0 µm as a function of the pump intensity, where *P*_*y*_ is the *y* component of the ponying vector, S1 and S2 are two distant cutting planes, as marked in (a).

Figure 1(a) shows the MQW-based PTI used for the proposed all-optical modulator. It is a honeycomb lattice of cylindrical pillars made of TiN/Al_2_O_3_ MQWs. The optical nonlinear PTI is designed to have two different topological modes depending on the unit geometry and its refractive index. The intensity-dependent refractive index of the MQWs is adapted from our previous works [33], see details in Supplementary Material Section 1. Without laser pumping, the MQWs are a lossy metal so that PTI in both unit geometries, i.e. the expanded one with a larger distance between adjacent cylindrical pillars *R*_1_ and the shrunken one with a smaller distance between adjacent cylindrical pillars *R*_2_, is a plasmonic structure. In this case, the surface plasmon polariton (SPP) mode is supported at the PTI surface, and its center wavelength is designed to located at the working wavelength around 2.0 μm in order to restrict the signal transmission (see the upper panel of Figure 1(b)). The signal transmission is allowed on the PTI surface under laser pumping (see the lower panel of Figure 1(b)), owing to the high-intensity low loss feature of the MQWs [33]. In the case of pumping, the expanded PTI is designed to have a nontrivial band structure, while the shrunken PTI has a trivial band structure; so, by adjoining the two PTI structures, a zigzag-shaped topological interface is formed where topologically protected pseudospin-dependent edge modes are enabled [36], which can efficiently carry the signal. Figure 1(c) shows the intensity dependence of the signal transmission: Around −21.3 dB without laser pumping while around −5.8 dB under pumping. Such a huge transmission contract (∼15 dB) is essential for an all-optical modulator, as demonstrated in what follows.

**Figure 2:**
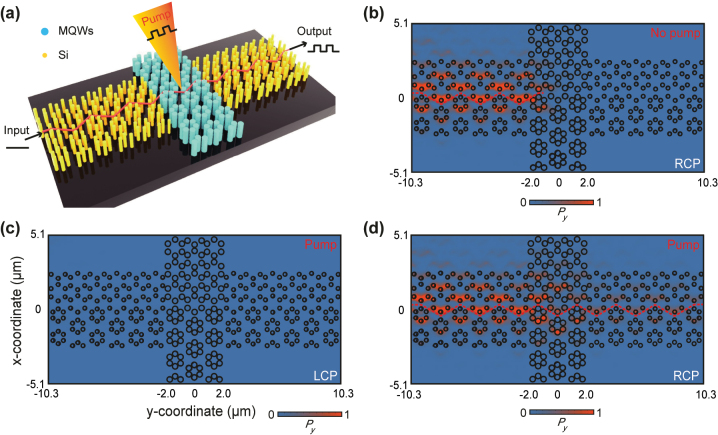
All-optical modulator with PTIs made of MQWs. (a) Schematic of the proposed MQW-based PTI all-optical modulator sandwiched by two Si topological waveguides. The input signal, guided by the input Si topological waveguide, undergoes modulation at the PTI modulator by a modulated pumping laser before entering the output Si topological waveguide. (b) *P*_*y*_-field distribution of the RCP signal light without laser pumping. (c, d) *P*_*y*_-field distribution of the LCP (c) and RCP (d) signal light under laser pumping, respectively.

Figure 2(a) shows the proposed PTI all-optical modulator, which is sandwiched by two Si topological waveguides. The two Si topological waveguides are designed to maximize the coupling efficiency at the interface to PTI (see details in Supplementary Material Section 3). Figure 2(b) and (d) show the simulation results of *P*_*y*_-field distribution of the right-circularly polarized (RCP) signal light at the wavelength of 2.0 µm without and with laser pumping, respectively, and *P*_*y*_ is the *y* component of the Poynting vector. In the absence of a pumping laser, the input signal light is localized at the PTI interface (Figure 2(b)), indicating that the signal is not allowed to pass the PTI modulator; in this case, the modulator is in the “OFF” state. It is in the “ON” state under laser pumping, and the input signal is then allowed to pass the zigzag-shaped topological interface of PTI, as shown in Figure 2(d). Therefore, these simulation results show clearly that the proposed PTI modulator can be used to efficiently modulate the RCP signal light by alternating the pumping laser. Moreover, the topological protection feature of PTI enables the modulator to completely suppress the transmission of the left circularly polarized (LCP) light, as shown in Figure 2(c), which thus prevents the backscattering of the RCP signal light. Further studies could explore the comparative back scattering properties of topological versus standard waveguide structures to provide a clearer understanding of the suppression mechanisms involved.

**Figure 3:**
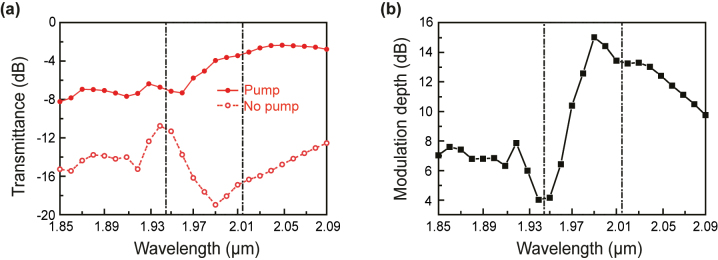
Performance of the proposed PTI all-optical modulator. (a) Wavelength dependence of the transmittance for the RCP signal light without and with laser pumping. (b) Wavelength dependence of the corresponding modulation depth. The dashed squares mark the band gap of the PTI.

For an all-optical modulator, the transmittance and modulation depth are two of the key performance metrics. Figure 3(a) summarizes the wavelength dependence of the transmittance for the RCP signal light with the proposed PTI all-optical modulator. Without laser pumping, the transmittance remains low in the wavelength range around 2.0 μm – due to the excitation of lossy SPP modes (Figure 1(b)) – with a minimum value at the wavelength of 1.99 μm. Upon laser pumping, the transmittance is significantly increased due to the optically induced metallic-to-dielectric transition of MQWs [33]. In this case, the topological edge modes are responsible for the signal light transmission at the zigzag-shaped topological interface (Figure 1(b)). The calculated modulation depth of the all-optical modulator is summarized in Figure 3(b). The modulation depth reaches 15 dB at the wavelength of 1.99 µm in the topological band gap where the optical backscattering is forbidden. Such a substantial transmittance contract is crucial for the functionality of all-optical modulators. Detailed calculation methods of transmittance and modulation depth are shown in Supplementary Material Section 4. In addition, based on our previous studies of MQWs [37], the modulation speed of the proposed modulator can reach the order of 100 fs, which corresponds to a modulation bandwidth up to the order of 10 THz.

**Figure 4:**
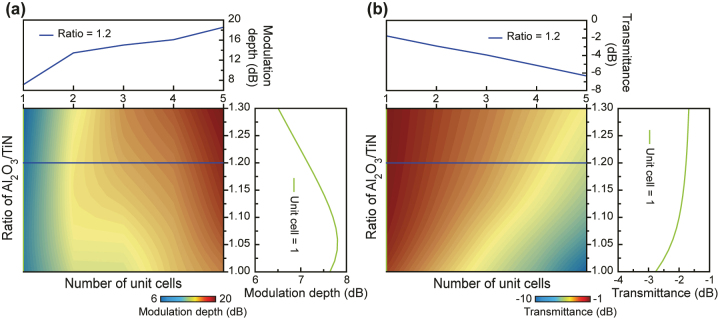
Dependence of the performance of the proposed PTI all-optical modulator. (a, b) Modulation depth (a) and transmittance (b) as functions of the number of unit cells and the thickness ratio of Al_2_O_3_ to TiN at the wavelength of 1.99 µm. The upper insets show the results at different numbers of unit cells when the Al_2_O_3_ to TiN thickness ratio is fixed to 1.2, while the results at different Al_2_O_3_ to TiN thickness ratios when only one unit cell is considered are shown in the lower insets.

PTI-based photonic devices are able to operate at small scales due to their topological nature [35], which is particularly advantageous for the development of compact and integrated optical circuits. To assess the performance of the proposed PTI all-optical modulator under different configurations, the dependence of the number of unit cells and the thickness ratio of Al_2_O_3_ to TiN in MQWs on the modulation depth and the transmittance is calculated at the wavelength of 1.99 µm and the results are summarized in Figure 4. It is evident that all the modulation depth is above 6 dB in the available unit cell range at various Al_2_O_3_ to TiN thickness ratios (Figure 4(a)), showing the high flexibility in the design of the proposed PTI all-optical modulator. Since a 3-dB modulation depth is sufficient for practical applications, the transmission efficiency becomes the dominant factor in designing the PTI all-optical modulator with a smaller footprint. As shown in Figure 4(b), the transmittance is increased as the Al_2_O_3_ to TiN thickness ratio increases and also as the number of unit cells decreases. As the consequence, the proposed PTI all-optical modulator can achieve a footprint down to 1.3 × 10 µm^2^ (i.e. only one unit cell) at the 6.5-dB modulation depth with a transmittance up to −1.68 dB (at the Al_2_O_3_ to TiN thickness ratio of 1.30). All-optical modulators with such a small footprint are suitable for the miniaturization of optical devices in integrated optical networks. It is worth noting that our work, which employs a circularly polarized beam for the PTI waveguide due to pseudo-time-reversal symmetry, can be extended to systems using a linearly polarized beam, such as quantum Hall photonic topological insulators.

## 3 Conclusion

In conclusion, we have presented an on-chip all-optical modulator with PTIs made of MQWs. The all-optical modulator has been designed to have a substantial modulation depth of 15 dB with a modulation bandwidth above THz in a tiny footprint of only 4 × 10 µm^2^. Such a high-performance all-optical modulator could facilitate the realization of large-scale dense photonic integrated circuits. In regards to the fabrication of the proposed MQW-based PTI all-optical modulator, the growth of high-quality MQWs is critical in the future works. The MQWs-elements consist of multiple pairs of ultrathin TiN and Al_2_O_3_ layers, which could be fabricated as the film-stack [32], [33]. Subsequently, the MQWs-element topological structures could be obtained through standard lithography processes such as focused ion beam milling and plasma etching [38]. To this end, MQWs with two-dimensional material heterostructures may be a better alternative, which could further improve the all-optical modulation performance [39], [40], [41].

## Supplementary Material

Supplementary Material Details
