# Supplementary material for: All-optical modulator with photonic topological insulator made of metallic quantum wells
Source: Nanophotonics. 2024 Jun 26;13(18):3575–80. doi: 10.1515/nanoph-2024-0197 (PMC11501365; doi:10.1515/nanoph-2024-0197)
Supplement: Supplementary file 1 — Supplementary Material Details [file j_nanoph-2024-0197_suppl_001.docx]

**Supplementary Material**

All-optical modulator with photonic topological insulator made of metallic quantum wells

**1. Intensity-dependent refractive index of MQWs**

**2. Detailed calculation methods of spectra for expanded and shrunken PTIs**

**3. Detailed design method of the Si topological waveguide**

**4. Detailed calculation methods of transmittance and modulation depth**

**Section 1. Intensity-dependent refractive index of MQWs**

The MQWs used in the proposed PTI all-optical modulator are formed by an Al_2_O_3_/TiN/Al_2_O_3_ sandwich structure and have been proven to have a giant optical Kerr nonlinearity [1]. In this work, the intensity-dependent complex refractive index of TiN is extracted from our previous experiment [1] and the refractive index of Al_2_O_3_ is taken from the reference [2]. Then, the optical property of the resultant MQWs can be calculated by effective medium theory (EMT) as follows [3]:

$\varepsilon_{\parallel}=\rho\varepsilon_{\mathrm{TiN}}+(1-\rho)\varepsilon_{\mathrm{Al}_{2}O_{3}}$, (1)

$\frac{1}{\varepsilon_{\perp}}=\frac{\rho}{\varepsilon_{\mathrm{TiN}}}+\frac{1-\rho}{\varepsilon_{\mathrm{Al}_{2}O_{3}}}$, (2)

$\rho=\frac{t_{\mathrm{TiN}}}{t_{\mathrm{TiN}}+t_{\mathrm{Al}_{2}O_{3}}}$, (3)

where $\varepsilon_{\parallel}$ and $\varepsilon_{\perp}$ are the parallel and vertical components of the permittivity of MQWs, respectively, and $\rho$ is the thickness ratio of TiN. Figure S1 shows the calculated intensity-dependent complex refractive index of the MQWs at the TiN to Al_2_O_3_ thickness ratio of 1:1.20.


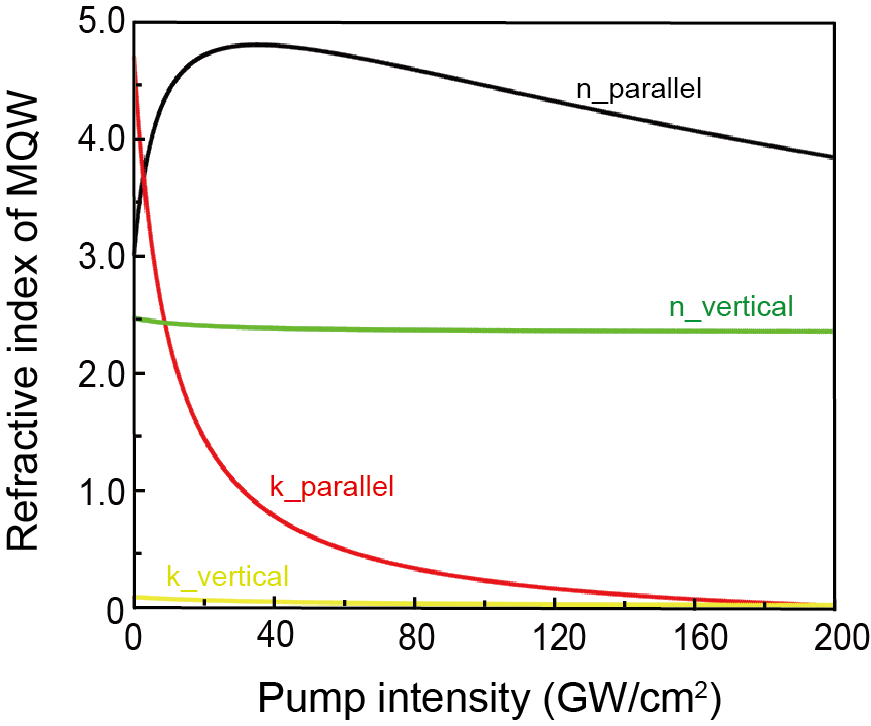


**Figure S1.** Intensity dependence of the complex refractive index of MQWs calculated at the wavelength of 2.0 µm and the thickness ratio of TiN to Al_2_O_3_ of 1:1.20.

**Section 2. Detailed calculation methods of spectra for expanded and shrunken PTIs**

In this section, we present a detailed description of the model created in COMSOL to perform the simulation of spectra for expanded and shrunken PTIs. Figures S2(a and b) show the unit geometries of the expanded and shrunken PTIs, respectively. In these geometries, the cylindrical pillars are made of MQWs, while the remaining parts are consisted of air. To optimize the Kerr nonlinearity of MQWs at the target operating wavelength of approximately 2.0 µm, the unit geometry constants are set to follows: the lattice period *a* = 1.58 µm, the diameter of the pillar *d* = 0.37 µm and its height *h* = 1.0 µm. The distance between adjacent cylindrical pillars *R* of two different unit geometries is set as *a*/*R*_1_ = 2.75 and *a*/*R*_2_ = 3.65 for the expanded and shrunken PTIs, respectively.

Figures S2(c and d) show the spectra of the expanded PTI before and after the topological transition point of 100 GW/cm^2^ (Figure 1(c)). There is a high absorptance peak corresponding to the SPP mode at the working wavelength around 2.0 µm where the signal transmission is restricted, whereas afterward, a distinct nontrivial band gap becomes evident so the signal transmission is allowed on the PTI surface.


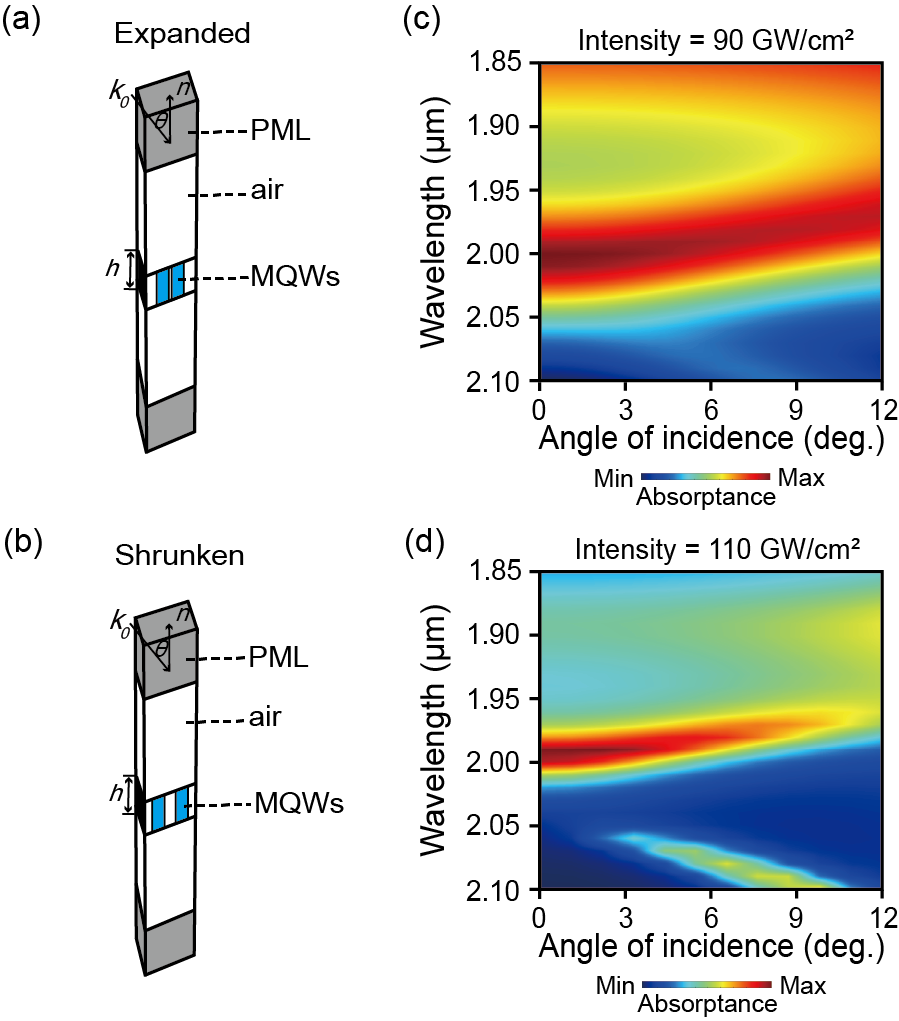


**Figure S2.** (a,b) Schematics of unit geometries for the expanded (a) and shrunken (b) PTIs made of MQWs. Perfectly matched layers (PMLs) are applied. (c,d) Absorptance as functions of the wavelength and the incidence angle (*θ*) of *p*-polarized incident light for the expanded PTI before (c) and after (d) the topological transition point of 100 GW/cm^2^.

**Section 3. Detailed design method of the Si topological waveguide**

In this part, we show the design method of the Si topological waveguide. Similar to the MQW PTI, the Si topological waveguide is formed by a honeycomb lattice of cylindrical pillars made of Si (Figures S3(a and b)). To match the zig-zag topological interface of the proposed PTI modulator, the unit geometry of the Si topological waveguide is set as follows: the lattice period *a*_0_ = 1.58 µm, the diameter of the pillars *d*_0_ = 0.24 µm and its height *h* = 1.0 µm. The distance between adjacent cylindrical pillars *R* of the expanded and shrunken Si PTIs is set as *a*_0_/*R*_1_ = 2.75 and *a*_0_/*R*_2_ = 3.65.

Figures S3(c and d) show the calculated photonic band structures of two distinct Si PTIs using the eigenfrequency module in COMSOL. There is a double degeneracy at the Diarc point, corresponding to the *p_±_* and *d_±_* states of *E_z_* field [4]. For the case of a smaller distance between adjacent cylindrical pillars, the lower (upper) photonic bands are occupied by the *p_±_* (*d_±_*) states (Figure S3(d)). As the distance between adjacent cylindrical pillars is increased, a band inversion takes place (Figure S3(c)) so that the expanded Si PTI has a nontrivial band structure, while the shrunken Si PTI takes a trivial band structure. Therefore, by adjoining the expanded and shrunken Si PTIs, a zigzag-shaped topological interface is formed where topologically protected pseudospin-dependent edge modes are supported.


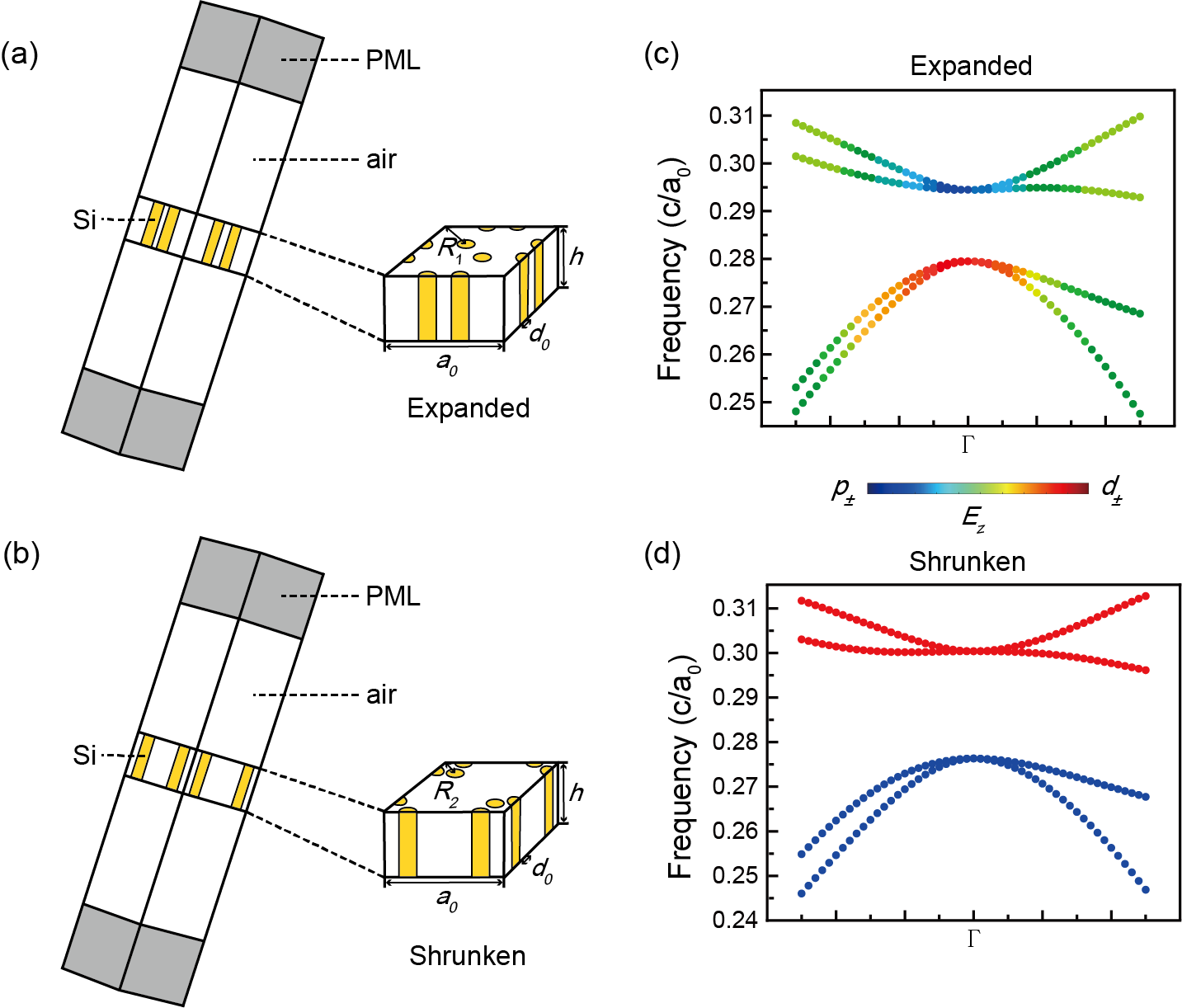


**Figure S3.** (a,b) Schematics of unit geometries of the expanded (a) and shrunken (b) PTIs made of Si. (c,d) Photonic band structures of the expanded (c) and shrunken (d) Si PTIs.

**Section 4. Detailed calculation methods of transmittance and modulation depth**

In this section, we show the calculated methodology used to determine the transmittance and modulation depth of the PTI all-optical modulator, as summarized in Figure S4. The transmittance is defined as *T* = *P_y_*__S1_/*P_y_*__S2_, where *P_y_* represents the *y* component of the Poynting vector, and S1 and S2 correspond to the ports of the input and output signals. The modulation depth is defined as *M* = *T*_Pump_ / *T*_No-pump_ with *T*_Pump_ and *T*_No-pump_ denoting the transmittance with and without laser pumping, respectively.


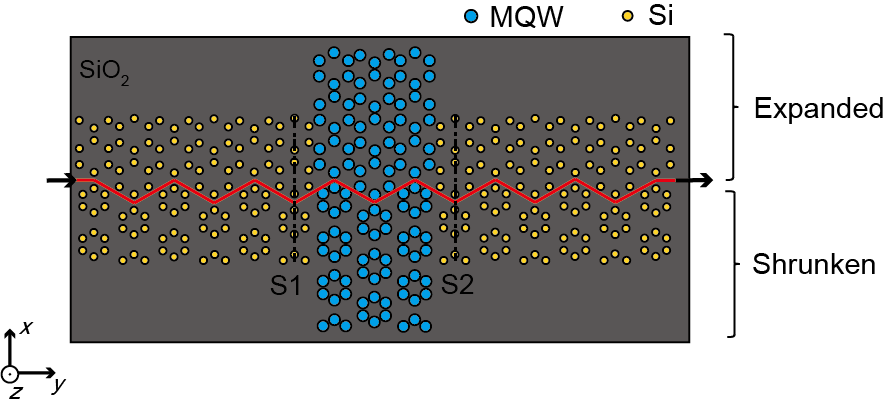


**Figure S4.** Top view of the proposed PTI all-optical modulator. Two distinct cutting planes S1 and S2 represent the ports of the input and output signals.

**References**

[1] H. Qian, S. Li, Y. Li, et al., "Nanoscale optical pulse limiter enabled by refractory metallic quantum wells," *Sci. Adv.*, vol. 6, p. eaay3456, 2020.

[2] I. H. Malitson, "Refraction and dispersion of synthetic sapphire," *J. Opt. Soc. Am.*, vol. 52, pp. 1377-1379, 1962.

[3] T. C. Choy, *Effective medium theory: principles and applications*, Oxford University Press, 2015.

[4] L.-H. Wu and X. Hu, "Scheme for achieving a topological photonic crystal by using dielectric material," *Phys. Rev. Lett.*, vol. 114, p. 223901, 2015.
